# Phenolic Acid Profiling, Antioxidant, and Anti-Inflammatory Activities, and miRNA Regulation in the Polyphenols of 16 Blueberry Samples from China

**DOI:** 10.3390/molecules22020312

**Published:** 2017-02-18

**Authors:** Xianming Su, Jian Zhang, Hongqing Wang, Jing Xu, Jiuming He, Liying Liu, Ting Zhang, Ruoyun Chen, Jie Kang

**Affiliations:** 1 State Key Laboratory of Bioactive Substance and Function of Natural Medicines, Institute of Materia Medica, Chinese Academy of Medical Sciences & Peking Union Medical College, No. 1 Xiannongtan Street, Beijing 100050, China; suxianming@imm.ac.cn (X.S.); wanghongqing@imm.ac.cn (H.W.); xujing@imm.ac.cn (J.X.); hejiuming@imm.ac.cn (J.H.); liuliying@imm.ac.cn (L.L.); rych@imm.ac.cn (R.C.); 2 Department of Bioengineering, Zhuhai Campus of Zunyi Medical University, Zhuhai 519041, China; jianzhang@hotmail.com; 3 Institute of Medical Information & Library, Chinese Academy of Medical Sciences & Peking Union Medical College, No. 3 Yabao Street, Beijing 100020, China; brendatingting@126.com

**Keywords:** Chinese blueberry, highbush (*Vaccinium corymbosum*), half-highbush (*Vaccinium corymbosum*/*Vaccinium anugustifolium*), antioxidant activity, anti-inflammatory activity, miRNA

## Abstract

To investigate the anti-atherosclerosis related mechanism of blueberries, the phenolic acids (PAs) content, antioxidant and anti-inflammatory activities, as well as the microRNA (miRNA) regulation of polyphenol fractions in blueberry samples from China were studied. Sixteen batches of blueberries including 14 commercialized cultivars (Reka, Patriot, Brigitta, Bluecrop, Berkeley, Duke, Darrow, Northland, Northblue, Northcountry, Bluesource, Southgood, O’Neal, and Misty) were used in this study. Seven PAs in the polyphenol fractions from 16 blueberry samples in China were quantified by high performance liquid chromatography/tandem mass spectrometry (HPLC/MS^2^). The antioxidant activities of blueberry polyphenols were tested by (1,1-diphenyl-2-picrylhydrazyl [DPPH]) assay. The anti-inflammatory (tumor necrosis factor-α [TNF-α] and interleukin-6 [IL-6]) activities of the polyphenol fractions of the blueberries were investigated by using lipopolysaccharide (LPS) induced RAW 264.7 macrophages. The correlation analysis showed that the antioxidant (1,1-diphenyl-2-picrylhydrazyl [DPPH]) and anti-inflammatory (tumor necrosis factor-α [TNF-α] and interleukin-6 [IL-6]) activities of the polyphenol fractions of the blueberries were in accordance with their PA contents. Although the polyphenol-enriched fractions of blueberries could inhibit the microRNAs (miRNAs) (miR-21, miR-146a, and miR-125b) to different extents, no significant contribution from the PAs was observed. The inhibition of these miRNAs could mostly be attributed to the other compounds present in the polyphenol-enriched fraction of the blueberries. This is the first study to evaluate the PAs content, antioxidant and anti-inflammatory activities, and miRNA regulation of Chinese blueberries.

## 1. Introduction

Small berries constitute important sources of potential health-promoting phytochemicals. These fruits are rich sources of polyphenols, such as phenolic acids (PAs), anthocyanins, and other flavonoids [1]. Among them, PAs constitute a large group of secondary plant products with an aromatic ring bearing one or more hydroxyl substituents [2]. Epidemiological and laboratory studies show a convincing link between the antioxidant and anti-inflammatory properties of plant-derived polyphenolic compounds and their health-promoting and/or disease-preventing effects, such as anti-atherosclerosis [3,4], anti-aging [5], and improvement of metabolic syndrome [6]. In our previous study, seven PAs identified in the serum of rats fed a 10% lowbush blueberry diet were found to promote bone growth [7], to show potential athero-protective effects [8], and to attenuate mammosphere formation [9], which suggested that PAs may be important in vivo bioactive compounds in blueberries. In this study, PAs were firstly analyzed and quantified in polyphenol fractions of blueberry cultivars from China by high performance liquid chromatography/tandem mass spectrometry (HPLC/MS^2^).

The blueberry is one of the few fruits native to North America. Highbush, lowbush, and rabbiteye blueberries are the three major species in the US market. They have all found their way into agricultural practices worldwide and are part of the cuisine in areas ranging from Asia to the Mediterranean. Blueberries have been found to contain extremely high levels of polyphenols, including anthocyanins, procyanins, flavonols, and PAs, resulting in antioxidant and anti-inflammatory effects in vitro/vivo that provide health benefits [10,11,12,13,14,15,16], such as the protective effect against atherosclerosis in the apoE^−/−^ mouse model [17,18]. However, the mechanism that links polyphenols and the atherosclerosis-protective effects in blueberry cultivars in China are rarely reported.

MicroRNAs (miRNAs) are a class of endogenous, small, noncoding RNAs involved in post-transcriptional gene repression. They play critical roles in several different physiological processes. Interfering with miRNA expression in the artery wall is a potential approach toward addressing atherosclerotic plaques and cardiovascular disease development [19,20]. Specific miRNAs, such as miR-21, miR-146, and miR-125, have been found to be responsible for vascular inflammation and disease [21,22,23].

Since the 1980s, many blueberry cultivars have been introduced in China due to the very few species of blueberry available there. However, up to now, not much research has been conducted on the blueberry’s PAs levels, antioxidant and anti-inflammatory activities, and inhibition of atherosclerosis-related miRNAs in different imported (commercialized) blueberry cultivars grown in China [24,25,26,27]. Many factors, including climatic conditions, growing locations, harvest seasons, and cultivars can affect the contents and the bioactivities of blueberries [28,29,30].

The aim of the present work was to quantify the PAs contents in the polyphenol fractions of different Chinese blueberry cultivars, and to study the relationship between PAs contents and antioxidant and anti-inflammatory activities, and the inhibition of atherosclerosis-related miRNAs of the blueberry polyphenol fractions.

## 2. Results and Discussion

### 2.1. PAs Were Identified in Polyphenol-Enriched Fractions of 16 Blueberry Samples

Sixteen fresh blueberry samples belonging to highbush (North and South high) (*V. corymbosum*) and half-highbush (*V. corymbosum*/*V. anugustifolium*) were obtained from Dandong, Liaoning Province (samples **1**–**4**, **6**, **9**, and **11**–**14**), or Lijiang, Yunnan Province (samples **5**, **7**, **8**, **10**, **15**, and **16**), in China. The information about blueberry species, cultivars, collection origin and time, and the companies are listed in Table 1.

**Figure 1:**
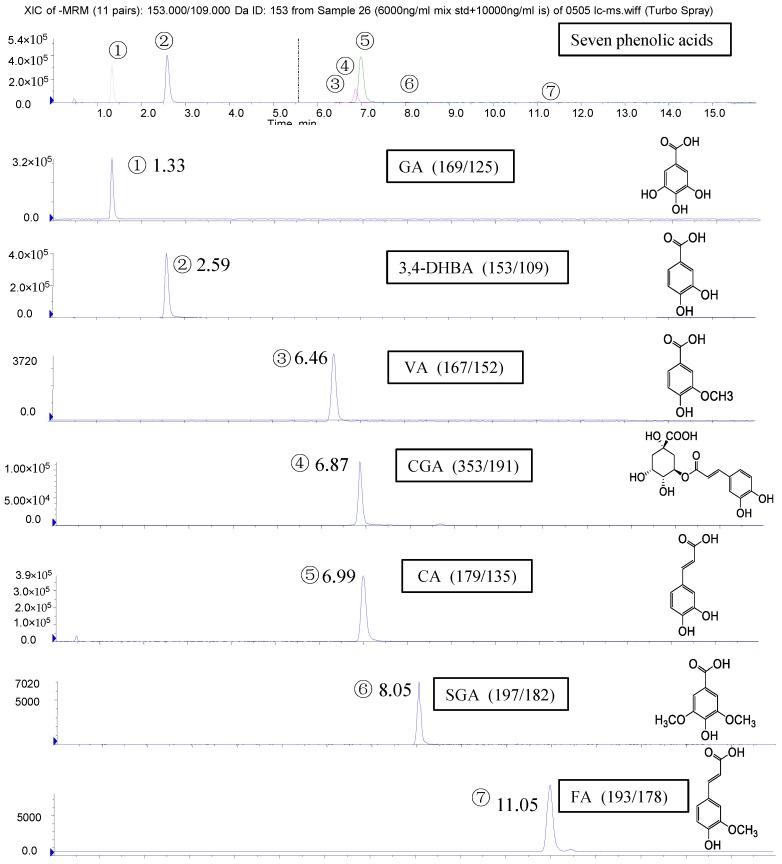
Chromatograms and structures of phenolic acids (PAs) quantified in blueberry polyphenol fractions with high-performance liquid chromatography coupled with electrospray ionization tandem mass spectrometry (HPLC/MS^2^). Please provide a clearer picture of all the Figures.

Identification of the PAs in the polyphenol-enriched fractions of the 16 blueberry samples was accomplished by comparing their retention time and MS data with those of the standards. A representative profile of the identified PAs and the structures of these PAs are both shown in Figure 1.

**Figure 2:**
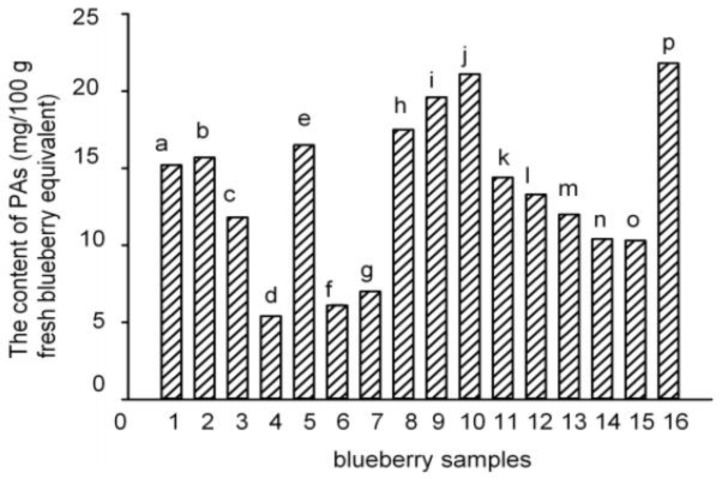
Total PA contents of the 16 blueberry polyphenol fractions. (The results have been expressed in terms of mg/100 g fresh blueberry equivalent, means ± SD, *n* = 3; columns with the different letters are significantly different, *p* < 0.05, *n* = 3).

Seven PAs (gallic acid (GA), 3,4-dihydroxybenzoic acid (3,4-DHBA), vanillic acid (VA), chlorogenic acid (CGA), caffeic acid (CA), syringic acid (SGA), and ferulic acid (FA)) were detected and quantified in the polyphenol-enriched fractions of seven blueberry samples (samples **5**, **8**, **10**, **12**, **13**, **15**, and **16**). The other nine blueberry samples (samples **1**–**4**, **6**–**7**, **9**, **11**, and **14**) contained only four to six PAs (Table 2). Samples **5**, **8**–**10**, and **16** had the highest levels of PAs (16.5–21.8 mg/100 g fresh blueberry equivalent), while samples **4**, **6**, and **7** had the lowest levels (5.4–7.0 mg/100 g fresh blueberry equivalent) (Figure 2). Among them, samples **5**, **8**, **10**, and **16** were identified to have the highest number and the highest content of PAs. Though seven PAs were all detected in samples **12**, **13**, and **15**, the contents of the total PAs were not very high. However, the PAs content of sample **9** was the third highest in the 16 samples, even though only six PAs were detected. Among the seven PAs, the level of CGA was the highest (84.4%–99.2% of total PAs), and the concentrations of the other six PAs were much lower (Table 2), which agreed with previous studies [2,10]. Therefore, the total content of PAs in the blueberries was mostly attributed to the amount of CGA.

A total of six blueberry samples (**5**, **7**, **8**, **10**, **15**, and **16**) were collected from Lijiang, Yunnan Province. Except for samples **7** and **15**, the other samples **5**, **8**, **10**, and **16** all had high PAs contents. Comparing the same cultivar of blueberries between two different locations (samples **4**–**5** and **9**–**10**), the blueberries from Lijiang, Yunnan Province had higher numbers and contents of PAs (Figure 2).

### 2.2. Antioxidant Activities of Polyphenol-Enriched Fractions of Blueberries

**Figure 3:**
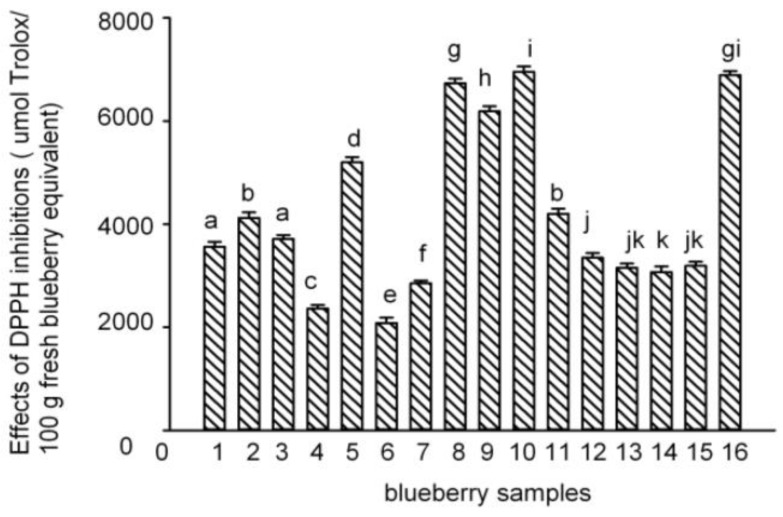
Effects of 1,1-diphenyl-2-picrylhydrazyl (DPPH) inhibition in the 16 blueberry polyphenol fractions. (The results have been expressed in terms of µmol Trolox/100 g fresh blueberry equivalent, means ± SD, *n* = 3; columns with the different letters are significantly different, *p* < 0.05, *n* = 3).

**Figure 4:**
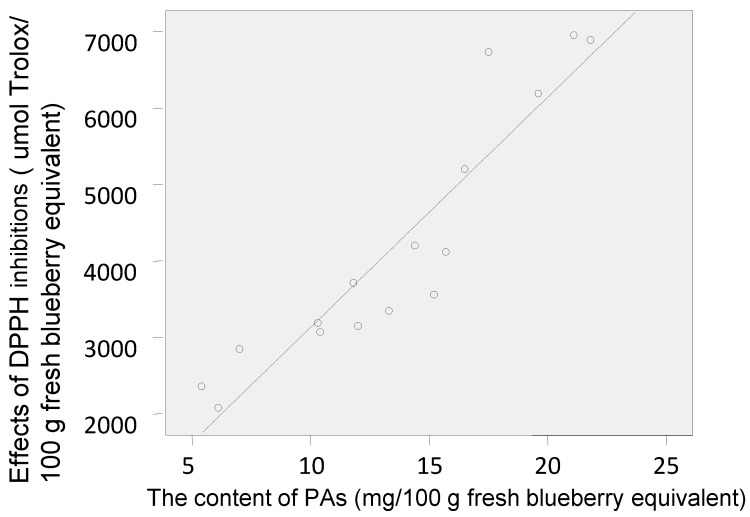
Pearson’s correlation between PAs content and antioxidant capacity (DPPH) of the 16 blueberry polyphenol fractions (Pearson’s correlation coefficient (*r*) = 0.926, significance at *p* = 0).

The antioxidant activities of the polyphenol-enriched fractions of the 16 blueberry samples were measured using the 1,1-diphenyl-2-picrylhydrazyl (DPPH) assay, which is one of the most widely used chemical-based antioxidant methods [31]. The results have been expressed in terms of µmol Trolox equivalent (TE) per 100 g fresh blueberry equivalent (IC_50 (Trolox)_/IC_50 (sample)_ × 100 g) [32]. The DPPH radical–scavenging activities of the polyphenol-enriched fractions of the 16 blueberry samples are shown in Figure 3. The PAs content showed a positive and direct correlation with the antioxidant capacity (DPPH) (Figure 4). Samples **5**, **8**–**10**, and **16** were more effective scavengers than the other samples, and except for sample **9**, all the other samples were from Lijiang, Yunnan Province; samples **4**, **6**, and **7** showed the weakest antioxidant activities (besides sample **7**, the other two were from Dandong, Liaoning Province).

### 2.3. Anti-Inflammatory Effects of Polyphenol-Enriched Fractions of Blueberries

**Figure 5:**
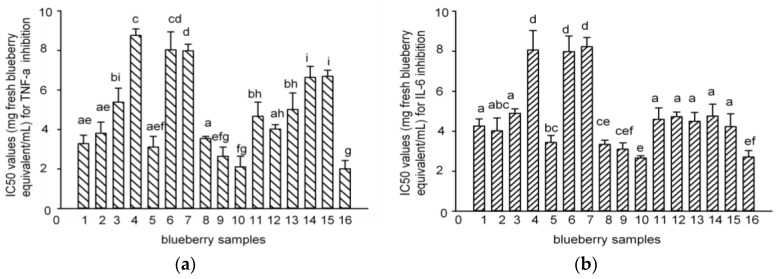
IC_50_ values of the 16 blueberry polyphenol fractions for TNF-α (**a**) and IL-6 (**b**) inhibition. (The results have been expressed in terms of IC_50_ (mg/100 g fresh blueberry equivalent, means ± SD, *n* = 3; columns with the different letters are significantly different, *p* < 0.05, *n* = 3).

**Figure 6:**
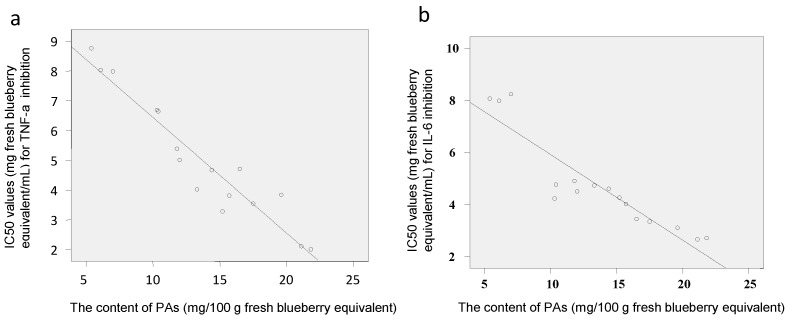
Pearson’s correlation between PAs contents and IC_50_ values of the 16 blueberry polyphenol fractions against inflammation, TNF-α (**a**) and IL-6 (**b**) (Pearson’s correlation coefficients (*r*) were −0.953 (**a**) and −0.916 (**b**), respectively, significance for both at *p* = 0).

Tumor necrosis factor-alpha (TNF-α) and interleukin-6 (IL-6) are well-known pro-inflammatory cytokines that modulate the expression of immune regulatory genes relevant in some critical illnesses including inflammatory related diseases [33]. RAW macrophages are widely used in the anti-inflammatory assay [34,35]. The IC_50_ values for the inhibition of lipopolysaccharides (LPS)-induced TNF-α and IL-6 production by polyphenol-enriched fractions in RAW 264.7 cells were 2.7–8.2 and 2.0–8.8 mg fresh blueberry equivalent/mL, respectively (Figure 5). The PAs content showed a negative and direct correlation with the IC_50_ values of the blueberry polyphenol fraction against inflammation (TNF-α and IL-6) (Figure 6), which meant that the samples with greater anti-inflammatory capacities matched the samples with the higher PA content. Samples **5**, **8**–**10**, and **16** that contained more PAs showed much stronger anti-inflammatory activities against IL-6 and TNF-α, while samples **4**, **6**, and **7** had less PAs that showed weaker activities. This result is in agreement with that of the antioxidant activities (DPPH).

### 2.4. miR-21, miR-146a, and miR-125b Inhibition in Polyphenol-Enriched Fractions of Blueberries

miR-21 and miR-146a are up-regulated in human atherosclerotic plaques, and miR-125b is involved in atherosclerosis obliterans [19,36]. Our results indicated that the polyphenol-enriched fractions of the 16 blueberry samples inhibited miR-21, miR-146a, and miR-125b to different extents (Table 3). Among them, miR-21 was the most inhibited by the blueberry polyphenol fractions, while miR-146a was the least inhibited. However, unlike the concordance between the PAs contents and the antioxidant and anti-inflammatory activities, the inhibitions of miR-21, miR-146a, and miR-125b were probably partially related to PAs, since no significant correlations were observed (Table 4). The miRNAs may be regulated by other compounds in the polyphenol-enriched fraction of the blueberries.

## 3. Materials and Methods

### 3.1. Chemicals and Reagents

GA, 3,4-DHBA, VA, CGA, CA, SGA, FA, benzoic acid-*d*_5_, DPPH, methanol (MeOH), DMSO, formic acid, phosphate-buffered saline (PBS), LPS from *Escherichia coli* O111:B4, and Histopaque 1077 were all obtained from Sigma-Aldrich (Milwaukee, WI, USA). Fetal bovine serum (FBS) was bought from Hyclone (Logan, UT, USA). Dulbecco’s Modified Eagle’s Medium (DMEM), RPMI-1640 culture medium, and 2′,7′-dichlorofluoresceindiacetate (DCFDA) were obtained from Invitrogen (Carlsbad, CA, USA).

### 3.2. Plant Materials

Sixteen fresh blueberry samples of highbush (North and South high) (*V. corymbosum*) and half-highbush (*V. corymbosum*/*V. anugustifolium*) were obtained from Dandong, Liaoning Province (samples **1**–**4**, **6**, **9**, and **11**–**14**) and Lijiang, Yunnan Province (samples **5**, **7**, **8**, **10**, **15**, and **16**), in China (Table 1). They were provided by two main Chinese blueberry companies, Jiawo and Pulan. The imported (commercialized) blueberry cultivars (Reka, Patriot, Brigitta, Bluecrop, Berkeley, Duke, Darrow, Northland, Northblue, Northcountry, Bluesource, Southgood, O’Neal, and Misty) were identified by Prof. Jiali An (Jiawo) and Prof. Lin Wu (Pulan). Blueberries were all collected during the harvest period (April and June) in 2015 (Table 1). After the fresh blueberries were harvested, they were frozen and stored at −20 °C until transport to the laboratory. The frozen samples were lyophilized and ground into powder; they were kept at −40 °C until analysis. The moister index of blueberry was calculated to be 86%.

### 3.3. Polyphenol-Enriched Extraction Procedure

The lyophilized blueberry powders were subjected to extraction with a mixed solvent (MeOH:H_2_O:acetic acid = 85:15:0.5); then, the extracts were purified using solid-phase extraction (SPE) cartridges with a 60-mL tube (Supelco, Milwaukee, WI, USA) filled with C-18 (Cosmosil 75 C18-PREP; Nacalai Tesque, Kyoto, Japan). The cartridge was equilibrated with 100 mL MeOH and washed with 100 mL 0.2% formic acid in H_2_O. The blueberry extract was loaded onto the column and washed with 100 mL 0.2% formic acid in H_2_O. Then, the polyphenol-enriched fraction was collected by washing the column with 100 mL 0.2% formic acid in MeOH. Finally, the sample was dried under nitrogen flow.

The dried sample was ready for DPPH assay, enzyme-linked immunosorbent assay, and the expression of miR-21, miR-125b, and miR-146a.

For the HPLC/MS^2^ analyses, the dried sample was reconstituted with 0.2 mg extract/mL in MeOH.

### 3.4. HPLC/MS^2^ Analysis

The HPLC/MS^2^ analysis was performed using an Agilent 1100 HPLC system including an autosampler, a binary pump, and a diode array detector (Agilent Technologies, Palo Alto, CA, USA), which was coupled to a 4000 Q TRAP mass spectrometer (Applied Biosystems, Foster City, CA, USA). Separation was carried on an Agilent Poroshell 120 SB-C18 column (50 × 3.0 mm, 2.7 μm) with a flow rate of 0.4 mL/min. The solvent consisted of (A) 0.2% (*v*/*v*) formic acid in water and (B) methanol. The 16 min gradient was as follows: 10%–14% B (0–2 min); 14%–18% B (2–4 min); 18%–25% B (4–6 min); 25%–27% B (6–8 min); 27%–30% B (8–10 min); 30%–35% B (10–15 min); 35%–35% B (15–16 min); and 10 min of re-equilibration of the column before the next run. The multi-reaction monitoring (MRM) mode scan was used for quantitation. A mass spectrometer equipped with an ESI-Turbo V source was operated in negative ion mode. The major parameters were optimized as follows: ion spray voltage, −4.5 kV; curtain gas (CUR), 50 psi; source temperature, 400 °C; nebulizing (GS1) and turbo spray gas (GS2), 30 and 50 psi, respectively. The entrance potential (EP), declustering potential (DP), collision energy (CE), and collision cell exit potential (CXP) were optimized individually with each standard. The postcolumn splitting ratio was 3:1. The analysis was controlled with Analyst v1.4.2 (Applied Biosystems, Forest City, CA, USA). The quantitative parameters for the PAs standards (1–7) and benzoic acid-*d*_5_(internal standard) (8) are shown below (Table 5).

### 3.5. DPPH Antioxidant Assay

The antiradical activity was determined using the DPPH method described by Grajeda-Iglesias [37]. Briefly, each sample was diluted in MeOH and then mixed with an equal volume of 0.25 mg/mL DPPH MeOH solution. The mixtures were added to a 96-well microplate and absorbance was read at 525 nm after 30min. Trolox (6-hydroxy-2,5,7,8-tetramethylchroman-2-carboxylic acid) was used as the internal control, and for the blank, the sample was substituted with MeOH. The concentration range of Trolox is 1, 10, 20, 40, 60, and 100 µM. Because the moister index of blueberry was calculated to be 86%, the results were expressed in terms of µmol Trolox equivalent (TE) per 100 g fresh blueberries (IC_50 (Trolox)_/IC_50 (sample)_ ×100 g) [32].

### 3.6. Enzyme-Linked Immunosorbent Assay Analysis of Interleukin-6 and Tumor Necrosis Factor-α Expression

The enzyme-linked immunosorbent assay was conducted based on the method previously reported [8]. RAW 264.7 cells (Invivogen, San Diego, CA, USA) were cultured in DMEM supplemented with penicillin (100 units/mL), streptomycin sulfate (100 µg/mL), l-glutamine (4 mM), and 10% (*v*/*v*) FBS (Hyclone, Logan, UT, USA). The cells (1 × 10^5^ cells/well) were then pretreated with various concentrations of polyphenol-enriched fractions of the 16 blueberry samples for 24 h before LPS (100 ng/mL) stimulation. After 18 h of LPS stimulation, the supernatant was collected. The TNF-α and IL-6 levels in the supernatant were determined with an enzyme-linked immunosorbent assay (ELISA) performed using Duoset ELISA kits (R&D, Minneapolis, MN, USA) according to the manufacturer’s instructions. The optical density was determined using a BMG Polar Star microplate reader (BMG Labtech, Durham, NC, USA) at 450 nm.

### 3.7. Analysis of miR-21, miR-125b, and miR-146a Expression

RAW 264.7 cells (Invivogen, San Diego, CA, USA) were cultured in DMEM supplemented with penicillin (100 units/mL), streptomycin sulfate (100 µg/mL), l-glutamine (4 mM) and 10% (*v*/*v*) FBS (Hyclone, Logan, UT, USA). The cells (1 × 10^5^ cells/well) were then pretreated with various concentrations of polyphenol-enriched fractions of the 16 blueberry samples for 24 h before LPS (100 ng/mL) stimulation. After 18 h of LPS stimulation, the supernatant was collected. For the detection of miRNA expression, the miScript Reverse Transcription Kit (Qiagen, Valencia, CA, USA) was used for cDNA synthesis. The miScript SYBR Green PCR Kit (Qiagen) was used in combination with a pair of miRNA-specific primers for the detection of mature miRNAs. RNU6B was used as an internal control.

### 3.8. Statistical Analysis

The post-hoc test was applied to perform ANOVA analysis, which was used to evaluate whether differences were significant. Pearson’s correlation coefficient was used to analyze the correlation between the content of phenolic acids and antioxidant and anti-inflammatory activities, as well as miRNA inhibitions. A value of *p* < 0.05 was considered as a significant difference. Statistical analyses were performed using SPSS 13.0 (IBM, New York, NY, USA).

## 4. Conclusions

To the best of our knowledge, this is the first report regarding the relationship between PAs contents and antioxidant and anti-inflammatory activities, and the inhibition of atherosclerosis-related miRNAs of blueberry polyphenol fractions. Sixteen blueberry samples belonging to 14 commercialized cultivars collected during the harvest time in China were used in this study. Correlation analysis showed that blueberries that had higher levels of PAs displayed stronger antioxidant and anti-inflammatory capacities. Most of the blueberry samples originating from Lijiang, Yunnan Province had better results than those from Dandong, Liaoning Province. The polyphenol-enriched fractions of the 16 blueberry samples inhibited three atherosclerosis related miRNAs, but the results did not reveal a positive relationship between the PAs contents and miRNAs inhibitions. Therefore, further study is required to determine which kinds of polyphenols play a role in the inhibition of miRNAs.

## Figures and Tables

**Table 1:** Species, cultivars, companies, collection origins, and collection time for the 16 blueberry samples.

No.	Species	Cultivars	Companies	Collection Origins	Collection Time
1	North high	Reka	Jiawo	Dandong, Liaoning Province	April 2015
2	North high	Patriot	Jiawo	Dandong, Liaoning Province	April 2015
3	North high	Brigitta	Jiawo	Dandong, Liaoning Province	April 2015
4	North high	Bluecrop	Jiawo	Dandong, Liaoning Province	April 2015
5	North high	Bluecrop	Pulan	Lijiang, Yunnan Province	June 2015
6	North high	Berkeley	Jiawo	Dandong, Liaoning Province	April 2015
7	North high	Duke	Pulan	Lijiang, Yunnan Province	June 2015
8	North high	Darrow	Pulan	Lijiang, Yunnan Province	June 2015
9	Half-high	Northland	Jiawo	Dandong, Liaoning Province	April 2015
10	Half-high	Northland	Pulan	Lijiang, Yunnan Province	June 2015
11	Half-high	Northblue	Jiawo	Dandong, Liaoning Province	April 2015
12	Half-high	Northcountry	Jiawo	Dandong, Liaoning Province	April 2015
13	South high	Bluesource	Jiawo	Dandong, Liaoning Province	April 2015
14	South high	Southgood	Jiawo	Dandong, Liaoning Province	April 2015
15	South high	O’Neal	Pulan	Lijiang, Yunnan Province	June 2015
16	South high	Misty	Pulan	Lijiang, Yunnan Province	June 2015

**Table 2:** Percentage (%) of each phenolic acid in the 16 blueberry polyphenol fractions.

No.	Phenolic Acids %						
	GA	3,4-DHBA	VA	CGA	CA	SGA	FA
1	-	-	1.19	98.36	-	0.34	0.12
2	-	-	0.68	98.98	-	0.28	0.06
3	-	-	2.07	97.24	-	0.25	0.44
4	-	-	2.46	96.63	-	0.61	0.29
5	0.06	0.18	3.00	95.67	0.68	0.28	0.14
6	-	-	5.64	93.51	-	0.72	0.13
7	-	0.50	2.60	95.38	0.25	1.27	-
8	0.27	0.51	2.21	95.50	0.92	0.33	0.27
9	0.09	-	1.21	98.20	0.22	0.20	0.09
10	0.63	0.67	5.14	90.93	1.52	0.42	0.69
11	-	0.04	2.35	97.22	-	0.34	0.04
12	0.34	0.43	1.76	90.45	6.40	0.37	0.25
13	0.03	0.13	0.02	99.23	0.16	0.33	0.11
14	-	-	1.71	97.59	0.24	0.38	0.08
15	1.61	1.76	8.34	84.39	2.26	0.84	0.81
16	0.41	0.61	2.43	94.48	1.18	0.52	0.37

**Table 3:** Inhibitory effects (%) of the polyphenol fraction of blueberries against miR-21, miR-125b, and miR-146a.

No.	microRNA (miRNA)		
	miR-21	miR-125b	miR-146a
1	49	34	12
2	50	26	13
3	52	18	14
4	50	30	13
5	53	27	16
6	43	28	15
7	53	31	15
8	48	32	12
9	52	28	15
10	60	37	22
11	61	41	23
12	55	33	21
13	41	38	6
14	46	34	7
15	44	23	8
16	50	36	13

**Table 4:** Pearson’s correlation coefficients (*r*) between PAs contents and inhibition rates of miR-21, miR-125b, and miR-146a of the 16 blueberry samples.

PAs	miRNA		
	miR-21	miR-125b	miR-146a
PAs content	0.405	0.271	0.281

No significance (*p* > 0.05).

**Table 5:** Quantitative parameters for the standards (**1**–**7**) and internal standard (**8**).

No.	Name	M.W.	Pairs	t_R_(min)	Equation ^a^	Linear Range (ng/mL)	RSD ^b^ (%)	LOD ^c^ (ng/mL)	LOQ ^d^ (ng/mL)
1	GA	170	169/125	1.33	y = 0.0044x − 123.297	50–6000	1.6	10	3
2	3,4-DHBA	154	153/109	2.59	y = 0.0025x − 171.664	50–6000	1.5	9	3
3	VA	168	167/152	6.46	y = 0.2240x − 63.617	500–6000	1.4	400	100
4	CGA	354	353/191	6.87	y = 0.0080x − 100.648	50–6000	1.3	30	10
5	CA	180	179/135	6.99	y = 0.0018x − 211.284	50–6000	0.7	20	6
6	SGA	198	197/182	8.05	y = 0.1513x − 27.866	100–6000	0.7	60	25
7	FA	194	193/178	11.05	y = 0.0856x − 19.518	200–6000	1.8	200	60
8	benzoic acid-*d*_5_	126	126/82	11.33			1.8		

^a^ y = concentration (ng/mL); x = peak area. ^b^ relative standard deviation (RSD). ^c^ limit of detection (LOD). ^d^ limit of quantity (LOQ).
